# Early Aspirin Discontinuation Following PCI for Acute Coronary Syndromes: A Systematic Review and Meta-Analysis of Reconstructed Individual Patient Data

**DOI:** 10.1016/j.jscai.2026.105456

**Published:** 2026-05-20

**Authors:** Vinícius Martins Rodrigues Oliveira, Lucas M. Barbosa, Ludimilla Pereira Tartuce, Robert Willian Anastácio Alcântara, Paulo Roberto Ferreira Tartuce Filho, Maria do Carmo Pereira Nunes, Humberto Graner Moreira, Bruno Ramos Nascimento, Deepak L. Bhatt

**Affiliations:** a Faculdade de Medicina, Universidade Federal de Goiás, Goiânia, Brazil; b Federal University of Minas Gerais, Belo Horizonte, Brazil; c Instituto de Cardiologia e Radiologia Intervencionista de Rio Verde, Rio Verde, Brazil; d Clínica Aurus, Oliveira, Brazil; e Einstein Hospital Israelita, São Paulo, Brazil; f Mount Sinai Fuster Heart Hospital, New York, New York

**Keywords:** acute coronary syndrome, dual antiplatelet therapy, meta-analysis, P2Y_12_ inhibitor, percutaneous coronary intervention

## Abstract

**Background:**

Dual antiplatelet therapy (DAPT) is well established for patients with acute coronary syndrome (ACS) undergoing percutaneous coronary intervention (PCI). Contemporary studies demonstrated that a shorter DAPT strategy may reduce bleeding, while preserving ischemic protection. However, optimal timing of aspirin discontinuation remains uncertain.

**Methods:**

We performed a systematic review and meta-analysis of randomized controlled trials comparing standard 12-month DAPT versus early aspirin discontinuation (≤1-2 months), following PCI for ACS, excluding studies with immediate aspirin withdrawal. We systematically searched PubMed, Embase, and Cochrane. Individual patient data were reconstructed from Kaplan–Meier (KM) curves. Hazard ratios (HRs) and their 95% CIs were estimated using Cox regression. All statistical analyses were performed using R software, version 4.5.0.

**Results:**

We included 5 randomized controlled trials comprising 12,984 patients; 6473 (49.8%) were randomized to early aspirin discontinuation. Short-term DAPT was associated with a reduction in the risk of bleeding (HR, 0.44; 95% CI, 0.34-0.55; *P* < .001), while preserving ischemic protection, with no differences in the risk of major adverse cardiac events (HR, 1.05; 95% CI, 0.83-1.32; *P* = .71). Stratified bleeding analyses demonstrated a reduction in major bleeding (HR, 0.40; 95% CI, 0.26-0.61; *P* < .01) and a reduction in clinically relevant bleeding (HR, 0.43; 95% CI, 0.34-0.55; *P* < .001).

**Conclusion:**

In trials evaluating aspirin discontinuation at approximately 1 month following PCI for ACS, transitioning to P2Y_12_ inhibitor monotherapy reduced bleeding while preserving ischemic protection in selected patients.

## Introduction

Dual antiplatelet therapy (DAPT) is recommended for patients with acute coronary syndrome (ACS) undergoing percutaneous coronary intervention (PCI) due to the increased risk of thrombotic complications following acute plaque rupture and stent implantation. The 2023 European Society of Cardiology guidelines recommend DAPT with aspirin and a potent P2Y_12_ inhibitor (P2Y_12_i) for at least 12 months following drug-eluting stent implantation in patients with ACS, with treatment duration individualized based on bleeding and ischemic risk assessment. The 2025 American College of Cardiology/American Heart Association guidelines recommend 12 months of DAPT with aspirin and ticagrelor or prasugrel for patients with ACS undergoing PCI, while also providing a class I, level A recommendation for ticagrelor monotherapy after 1 month of DAPT in patients at high bleeding risk as an alternative de-escalation strategy.^1^^,^^2^

The optimal duration of DAPT requires balancing the prevention of thrombotic events (efficacy) against bleeding complications (safety). While prolonged DAPT may demonstrate benefit regarding ischemic events, it increases bleeding risk, which is particularly harmful beyond the initial post-PCI period when endothelialization is largely complete. Recent randomized controlled trials and 1 large meta-analysis have demonstrated that shorter DAPT duration following PCI in patients with ACS may reduce bleeding events without significantly increasing ischemic complications.^3^, ^4^, ^5^

Contemporary studies have examined various aspirin discontinuation strategies, with some trials evaluating discontinuation after 1 to 3 months and others, including the recently published NEO-MINDSET (Percutaneous Coronary Intervention Followed by Monotherapy Instead of Dual Antiplatelet Therapy in the Setting of Acute Coronary Syndromes) and STOPDAPT-3 (Short and Optimal Duration of Dual Antiplatelet Therapy-3) trials,^6^ implementing immediate aspirin discontinuation following PCI, with continuation of a potent P2Y_12_i monotherapy.^7^^,^^8^ These approaches are based on emerging evidence that aspirin may contribute disproportionately to bleeding risk relative to its antithrombotic benefit in the postacute phase following PCI, when the thrombotic risk seems to consistently decrease, especially with the biocompatibility of newer generation drug-eluting stents and potency of newer antiplatelets.

Given the heterogeneity in study designs and conflicting evidence regarding optimal DAPT duration, we conducted a systematic review and meta-analysis of individual patient-level reconstructed time-to-event data to evaluate the efficacy and safety of aspirin discontinuation at approximately 1 month following PCI in patients with ACS.

## Materials and methods

Our study was performed in accordance with the Cochrane Handbook for Systematic Reviews of Interventions and the Preferred Reporting Items for Systematic Reviews and Meta-Analysis (PRISMA) guidelines^9^^,^^10^ (Supplemental Methods 1 and 2). The protocol was prospectively registered on PROSPERO (CRD420251144691). The data that support the findings of this study are available from the corresponding author for replication upon reasonable request. The research reported has adhered to the relevant ethical guidelines, and patient consent was not required for this study.

We systematically searched PubMed, Embase, and Cochrane databases on September 2025. Two authors (V.M.R.O. and L.M.B.) independently assessed all records retrieved, and a decision regarding inclusion was achieved through consensus between them. The complete search strategy for each database is presented in Supplemental Methods 3.

Studies were included if they met the following criteria: (1) randomized controlled trials (2) comparing standard 12-month DAPT vs DAPT of approximately 1 to 2 months’ duration, followed by a P2Y_12_i monotherapy—namely prasugrel, ticagrelor, or clopidogrel (3) following PCI for ACS using contemporary drug-eluting stents, which reported the composite ischemic end point or the bleeding end point within a Kaplan–Meier (KM) curve. Studies discontinuing DAPT at time zero (upon randomization or right after PCI) were not included.

### End points

Our primary efficacy end point was major adverse cardiac events (MACE), in most studies defined as a composite of all-cause or cardiovascular disease–related mortality, myocardial infarction, target-vessel revascularization, and stent thrombosis. Our primary safety end point was bleeding—Bleeding Academic Research Consortium (BARC) types 3 or 5 (applied whenever possible); thrombolysis in myocardial infarction (TIMI) major, with clinically relevant bleeding defined as BARC types 2, 3, or 5; and TIMI major or minor bleeding. The secondary end points were net adverse clinical event (NACE), defined as a composite of all-cause death, myocardial infarction, stroke, or major bleeding, and the components of the composite end points: all-cause mortality, myocardial infarction, stroke, target-vessel revascularization, stent thrombosis (as per-study definition), major bleeding, and clinically relevant bleeding. Detailed end point definitions for each included study and the main inclusion and exclusion criteria are provided in Supplemental Tables S1 and S2.

### Quality assessment

Quality assessment was conducted using Cochrane Risk of Bias 2 tool.^11^ The risk of bias assessment was performed independently by 2 authors. Disagreements were resolved by consensus between authors. The Grading of Recommendations Assessment, Development, and Evaluation (GRADE), ranging from high, moderate, low, to very low, was applied for rating the certainty or quality of the evidence gathered from the systematic review and meta-analysis.^12^^,^^13^

### Statistical analysis

We obtained individual patient data (IPD) by reconstructing them from KM curves available in the published studies. The IPDfromKM package in R software (R Foundation for Statistical Computing) was used to extract time and survival probability coordinates from these curves, with this process carried out separately for intervention and control groups. Subsequently, IPD reconstruction was accomplished by integrating number at risk values or total patient counts alongside cumulative event numbers.^14^, ^15^, ^16^

Using the reconstructed data, cumulative event rates for the primary efficacy and safety outcomes were calculated. This comparison between 12-month DAPT and abbreviated DAPT (∼1 month) used pooled trial data and KM estimation techniques. For the end points of MACE, bleeding, and NACE, for which KM curves were available, reconstructed IPD from all trials were pooled in a 1-stage stratified Cox proportional hazards model (Surv[time, status] ∼ treatment + strata[trial]), where stratification by trial allowed each study its own baseline hazard function while assuming a common treatment effect across trials. For outcomes for which KM curves were not available, published hazard ratios (HRs) were pooled using a DerSimonian-Laird random-effects inverse-variance meta-analysis, with heterogeneity quantified by *I*^2^ and Cochran *Q*.^14^^,^^15^^,^^17^ A sensitivity analysis was conducted excluding trials that used clopidogrel as the P2Y_12_i or excluding this group from the included trials, when separate KM curves were available.

The proportional hazards assumption was assessed using Schoenfeld residuals test. For analyses where this assumption was violated (*P* < .05), restricted mean survival time analysis was performed, providing the mean event-free survival time and its difference between groups.^18^^,^^19^ All analyses were conducted using the survival, survminer, and survRM2 packages.

The number needed to treat (NNT) was calculated based on the absolute risk difference observed at 330 days postrandomization. This metric reflects the number of patients who would need to receive the intervention for 1 additional patient to avoid experiencing an event, providing a clinically interpretable measure of treatment effect magnitude.^16^^,^^20^

### Statistical analyses of secondary end points

Secondary outcomes were analyzed using a DerSimonian-Laird random-effects inverse-variance model to pool HRs and 95% CIs across studies. Individual trial results and pooled estimates were displayed through forest plots. We assessed between-study heterogeneity with Higgins’ *I*^2^ statistic, applying the Cochran Q test for statistical evaluation. Statistical significance was defined as *P* < .05 for all comparisons between treatment strategies.

## Results

**Figure 1:**
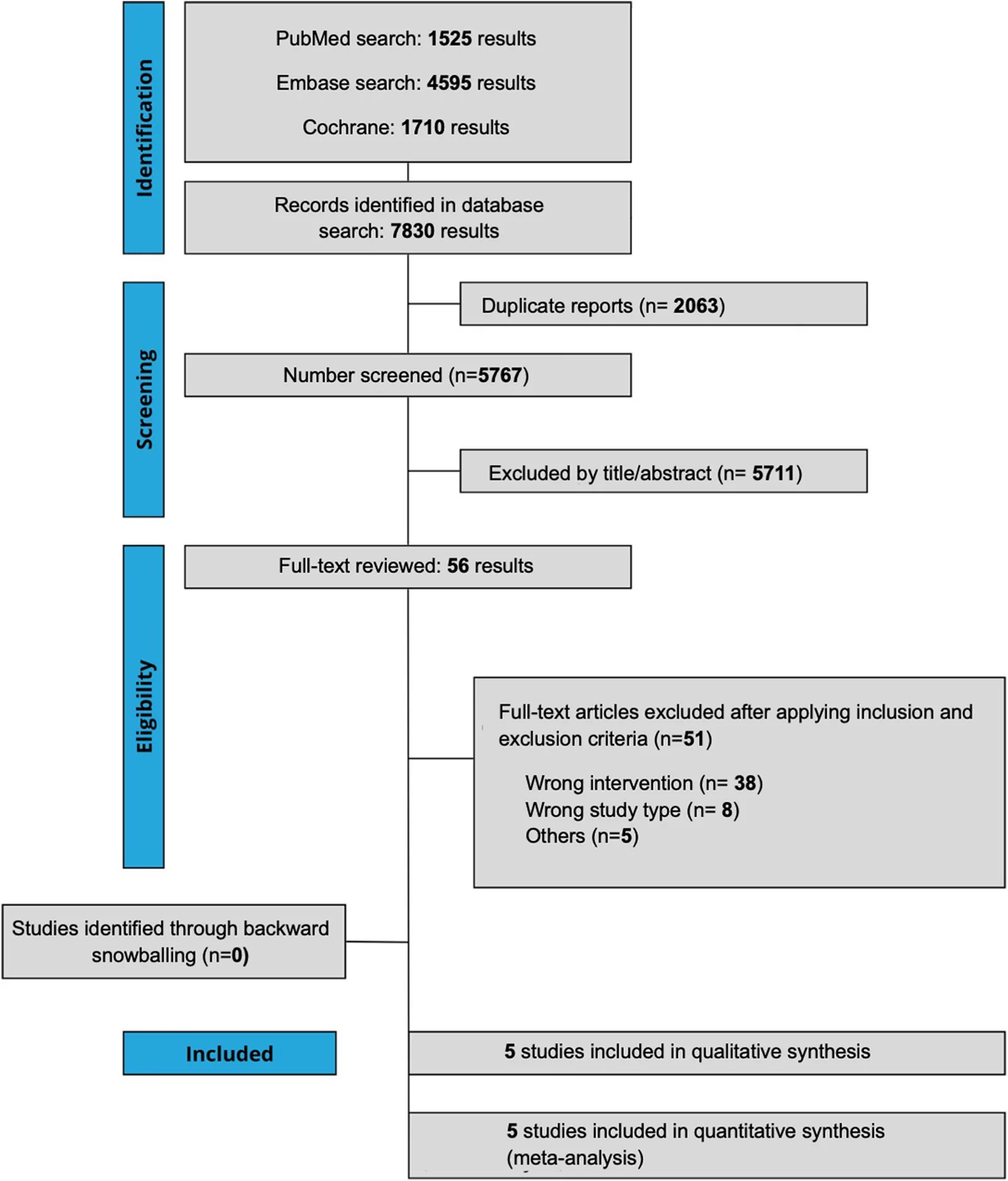
**PRISMA flow diagram.** PRISMA, Preferred Reporting Items for Systematic Reviews and Meta-Analysis.

Our search yielded 7830 results, ultimately including 5 RCTs comprising 12,984 patients, of whom 6473 (49.8%) were randomized to P2Y_12_i monotherapy^3^^,^^4^^,^^21^, ^22^, ^23^ (Figure 1). The mean age of participants ranged from 60.8 to 66.8 years, with 10,249 (79%) being males. The included trials varied regarding antiplatelet protocols: T-PASS^4^ and ULTIMATE-DAPT^3^ used ticagrelor, 4D-ACS^21^ used prasugrel, STOPDAPT-2 ACS^23^ used clopidogrel, while TARGET-FIRST^24^ used either clopidogrel, prasugrel, and ticagrelor. Three included trials discontinued aspirin after 1 month,^3^^,^^21^^,^^24^ while the STOPDAPT-2 ACS^23^ trial allowed aspirin withdrawal between 1 and 2 months (median, 39 days). The T-PASS study discontinued aspirin before 1 month (median, 16 days), and immediate discontinuation was not allowed.^4^ Detailed information about the included trials is provided in Table 1.^3^^,^^4^^,^^21^, ^22^, ^23^

**Table 1:** Baseline characteristics of the included studies

Study	Comparison	Sample size	Mean age, y	Female sex, %	Diabetes, %	STEMI, %	NSTE-ACS, %	Prior PCI, %	P2Y_12_i	Design	Follow-up period
ULTIMATE DAPT,^3^ 2024	1 mo of DAPT, followed by P2Y_12_ inhibitors alone vs DAPT for an additional 11 mo	3400	62	25.6	31.6	27.9	72.1	10.1	100% Ticagrelor	Placebo-controlled RCT	12 mo
T-PASS,^4^ 2024	<1 mo of DAPT (median, 16 d), followed by P2Y_12_ inhibitors alone vs DAPT up to 12 mo	2850	61	16.7	29.1	40.3	59.7	6.5	100% Ticagrelor	Open-label RCT	12 mo
4D-ACS,^21^ 2025	1 mo of DAPT, followed by P2Y_12_ inhibitors alone vs DAPT for an additional 11 mo	656	60.8	17.4	31.7	32.8	30.6	23	100% Prasugrel	Open-label RCT	12 mo
TARGET-FIRST,^22^ 2025	1 mo of DAPT, followed by P2Y_12_ inhibitors alone vs DAPT for an additional 10 mo	1942	61	21.6	14.4	50.4	49.6	6.3	74% Ticagrelor, 20.8% prasugrel, and 5.2% clopidogrel	Open-label RCT	12 mo
STOPDAPT-2 ACS,^23^ 2022	1 to 2 mo of DAPT (median, 39 d) followed by P2Y_12_ inhibitors alone vs 12 mo of DAPT	4136	66.8	20.7	29.7	73.7	26.3	10.3	100% Clopidogrel	Open-label RCT	12 mo

4D-ACS, The effect of short Duration of DAPT followed by Dose reduction after Implantation of DCS in ACS patients; DAPT, dual antiplatelet therapy; NSTE-ACS, non–ST-elevation acute coronary syndrome; PCI, percutaneous coronary intervention; STEMI, ST-elevation myocardial infarction; STOPDAPT-2 ACS, Short and Optimal Duration of Dual Antiplatelet Therapy-2 Study for the Patients With Acute Coronary Syndrome; TARGET-FIRST, Evaluation of a Modified Antiplatelet Therapy Associated With Low-dose DES Firehawk in Acute Myocardial Infarction Patients Treated With Complete Revascularization Strategy; T-PASS, Ticagrelor Monotherapy in Patients Treated With New-Generation Drug-Eluting Stents for Acute Coronary Syndrome; ULTIMATE-DAPT, Comparison of 1 Month vs 12-month Dual Antiplatelet Therapy After Implantation of Drug-Eluting Stents Guided by Either Intravascular Ultrasound or Angiography in Patients With Acute Coronary Syndrome.

**Figure 2:**
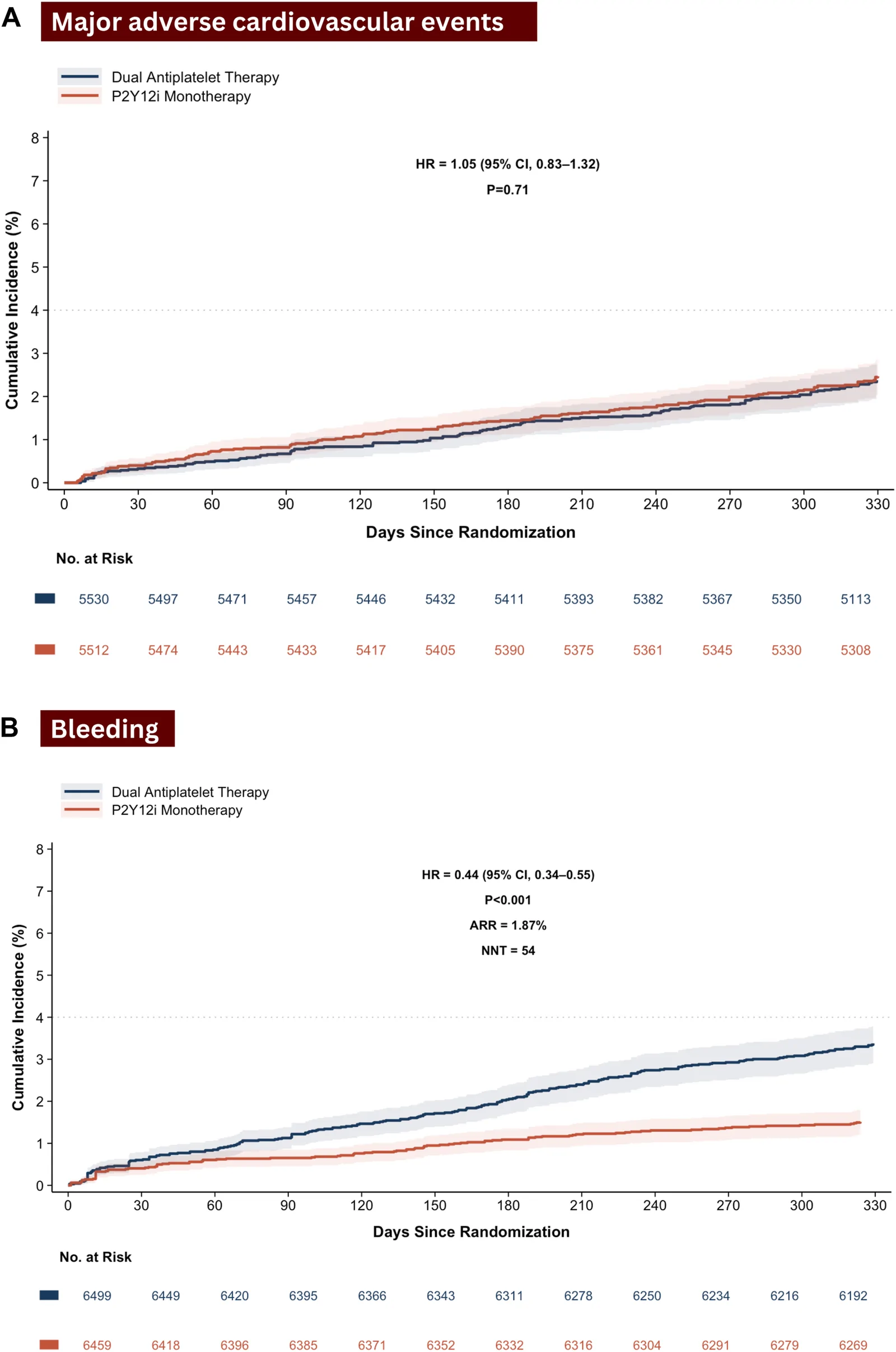
**Reconstructed Kaplan–Meier curve for major adverse cardiovascular events (A) and bleeding (B).** HR, hazard ratio; NNT, number needed to treat.

Patients randomized to the early discontinuation of aspirin were found at similar risk for the primary efficacy end point of MACE (HR, 1.05; 95% CI, 0.83-1.32; *P* = .71) (Figure 2A). The proportional hazards assumption was satisfied based on Schoenfeld residuals testing (*P* = .37). Landmark analyses were consistent with the overall results, showing no significant between-group differences during the first 30 days after randomization (HR, 1.23; 95% CI, 0.66-2.29; *P* = .52) (Supplemental Figure S1A) or from day 30 onward (HR, 1.01; 95% CI, 0.78-1.31; *P* = .94) (Supplemental Figure S1B).

For the primary safety end point of bleeding, early aspirin discontinuation was associated with a significantly lower risk than the standard 12-month DAPT. Due to evidence of nonproportional hazards (Schoenfeld test *P* = .015), we report the restricted mean survival time as the primary measure of treatment effect for this end point. At 330 days, patients in the monotherapy group had a mean of 3.06 additional bleeding-free days compared with DAPT (95% CI, 1.91-4.21), with a corresponding absolute risk reduction of 1.87% and a NNT of 54 to prevent 1 bleeding event (Figure 2B). The Cox model, reported for completeness, yielded an HR of 0.44 (95% CI, 0.34-0.55; *P* < .001). Landmark analyses showed no significant between-group difference during the first 30 days after randomization (HR, 0.67; 95% CI, 0.41-1.10; *P* = .11) (Supplemental Figure S2A), whereas from day 30 onward, P2Y_12_i monotherapy was associated with a significant reduction in bleeding events (HR, 0.39; 95% CI, 0.30-0.52; *P* < .001) (Supplemental Figure S2B).

**Figure 3:**
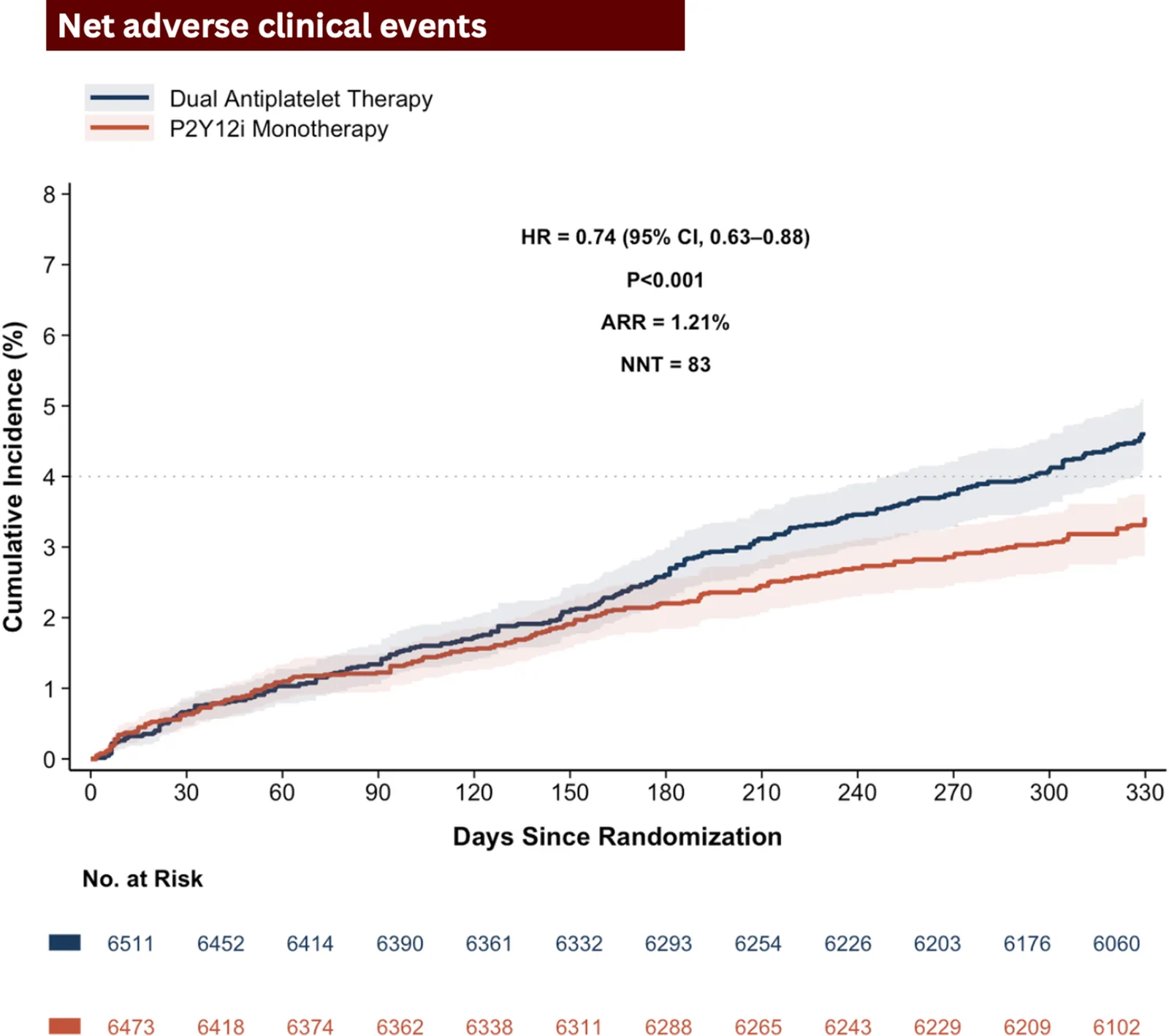
**Reconstructed Kaplan–Meier curve for net adverse clinical events.** HR, hazard ratio; NNT, number needed to treat.

Furthermore, early aspirin cessation reduced the risk of NACE (HR, 0.74; 95% CI, 0.63-0.88; *P* < .001; NNT, 83; absolute risk reduction, 1.21%) (Figure 3). Schoenfeld test confirmed proportional hazards assumption (*P* = .074).

Our sensitivity analysis restricted to trials using harmonized definitions for major bleeding (BARC types 3 or 5) demonstrated a consistent benefit of early aspirin discontinuation (HR, 0.40; 95% CI, 0.26-0.61) (Supplemental Figure S3). Similarly, the sensitivity analysis for clinically relevant bleeding restricted to trials defining this end point as BARC types 2, 3, or 5 also demonstrated a consistent benefit of P2Y_12_i monotherapy (HR, 0.43; 95% CI, 0.34-0.55) (Supplemental Figure S4).

Moreover, sensitivity analyses excluding the STOPDAPT-2 ACS study, which included only clopidogrel as P2Y_12_i, demonstrated a consistent reduction in the risk of bleeding (HR, 0.40; 95% CI, 0.31-0.53; *P* < .001) (Supplement Figure S5A), while showing no significant differences for the end point of MACE (HR, 0.88; 95% CI, 0.66-1.17; *P* = .38) (Supplemental Figure S5B).

Analysis of individual components of the primary efficacy composite end point revealed no significant differences between groups for all-cause death (HR, 1.26; 95% CI, 0.86-1.84; *P* = .23), myocardial infarction (HR, 1.22; 95% CI, 0.76-1.95; *P* = .41), stent thrombosis (HR, 1.22; 95% CI, 0.48-3.12; *P* = .67), stroke (HR, 0.96; 95% CI, 0.64-1.44; *P* = .83) or target-vessel revascularization (HR, 0.85; 95% CI, 0.59-1.20; *P* = .35), as shown in Supplemental Figure S6.

In our exploratory subgroup analyses for the end point of MACE, we found no significant interaction regarding ACS presentation type (ST-elevation myocardial infarction/non–ST-elevation myocardial infarction), with a p-value for subgroup differences of 0.25 (Supplemental Figure S7A). Similarly, no significant subgroup interaction was observed for the presence of diabetes mellitus (*P* = 0.47) (Supplemental Figure S7B). Regarding multivessel disease status, no significant subgroup interaction was detected (*P* = 0.15) (Supplemental Figure S7C).

Additionally, subgroup analyses for the bleeding end point revealed no significant interactions across clinical subgroups. No meaningful effect modification was identified when stratified by ACS presentation type (ST-elevation myocardial infarction/non–ST-elevation myocardial infarction; *P*_interaction_ = .099) (Supplemental Figure S8A), diabetes mellitus status (*P* = 0.69) (Supplemental Figure S8B), or multivessel disease (*P* = 0.90) (Supplemental Figure S8C).

In sensitivity analyses, we pooled the HRs using a random-effects inverse-variance method for the end points analyzed through KM reconstruction. The results were consistent with the IPD-based estimates for MACE (HR, 1.03; 95% CI, 0.76-1.40; *P* = .82) (Supplemental Figure S9A), bleeding (HR, 0.42; 95% CI, 0.33-0.54; *P* < .0001) (Supplemental Figure S9B), and NACE (HR, 0.70; 95% CI, 0.50-0.99; *P* = .04) (Supplemental Figure S9C; Supplemental Table S3). Of note, the TARGET-FIRST trial did not report an HR for NACE but provided a KM curve, which was reconstructed for inclusion in the IPD analysis. The risk of bias assessment showed the ULTIMATE-DAPT trial at low risk of bias and 4 studies with some concerns (Supplemental Figure S10).

### GRADE

The certainty of evidence regarding early aspirin discontinuation following PCI for ACSs was evaluated using the GRADE approach and was consistently rated as moderate for the end points of MACE, bleeding, and NACE, and as high for the end points of major bleeding (BARC 3 or 5) and clinically relevant bleeding (BARC 2, 3, or 5) (Supplemental Table S4). The primary limitation across end points was serious indirectness, as outcome definitions varied across trials, particularly for MACE and bleeding events (BARC and TIMI classifications).

For MACE, despite a large sample size of 11,042 patients from 4 RCTs with no concerns regarding risk of bias, inconsistency, or imprecision, the heterogeneous end point definitions reduced certainty to moderate. For the end point of bleeding, certainty remained moderate because of varying definitions. Similarly, NACE showed favorable effects (HR, 0.74), again limited by end point heterogeneity. Major bleeding (BARC 3 or 5) and clinically relevant bleeding (BARC 2, 3, or 5) were considered as end points with high certainty.

Overall, while all outcomes were derived from well-conducted RCTs with no serious methodological concerns, the variability in outcome definitions across studies limits the certainty of evidence. Nevertheless, findings consistently suggest that early aspirin discontinuation reduces bleeding risk without increasing ischemic events.

## Discussion

This meta-analysis of reconstructed individual patient-level data demonstrates that early aspirin discontinuation at approximately 1 month, and no longer than 2 months, following PCI in patients with ACS achieves a favorable balance between thrombotic and bleeding risks. Our analysis of 12,984 patients shows that transitioning to P2Y_12_i monotherapy after approximately 1 month of DAPT reduces bleeding events and NACE without compromising ischemic protection in selected patients.

These results, with a novel individual patient-level data reconstruction approach for this subject, contribute to the evolving paradigm regarding optimal antiplatelet management following ACS. Our findings support that approximately 1 month of DAPT, as evaluated in the included trials, may be sufficient before transitioning to P2Y_12_i monotherapy in selected patients—notably those at high bleeding risk—preserving ischemic benefits while reducing bleeding complications—at least in patients with ACS at the lower end of the ischemic risk spectrum. However, the scope of evidence and the number of trials available to date assessing this DAPT time range suggest individualized and multifactorial decision making by the Heart Team would be most appropriate, notably considering the ischemic risk, anatomical complexity, and bleeding hazard.

The clinical implications of these findings are particularly relevant when considering recent trial evidence examining various aspirin discontinuation strategies. The recently published NEO-MINDSET trial provided important insights regarding the safety of different aspirin withdrawal timepoints. Among patients who underwent successful PCI for ACS, immediate aspirin withdrawal (within 96 hours), followed by potent P2Y_12_i monotherapy was not found to be noninferior regarding the composite end point of death or ischemic events at 12 months,^25^ with an excess of ischemic events concentrated in the first 30 days. Moreover, in the recent STOPDAPT-3 trial, with a similar design, the very early aspirin-free strategy using prasugrel compared with DAPT failed to demonstrate superiority for major bleeding within 1 month after PCI but was noninferior for cardiovascular events.^6^ Thus, neither trial supported a very early aspirin-free strategy because of conflicting evidence around efficacy and risk concerns.

The prothrombotic state in the first weeks after an acute coronary event indeed justifies the need for an enhanced degree of platelet inhibition, which is achieved with the combination of 2 antiplatelet agents acting synergistically on key pathways of platelet activation. The mechanistic rationale for these temporal differences likely relates to the dynamic nature of thrombotic risk following ACS and stent implantation.^26^ However, the technical evolution of PCI, with newer generation drug-eluting stents, with thin struts and biocompatible polymers, or nonpolymeric designs,^27^ along with modern and optimized implantation techniques—including intravascular imaging^28^—have consistently decreased the risk of thrombotic events. In addition, the development of potent P2Y_12_i in the past 2 decades led to a reevaluation of the paradigm of long-term DAPT following PCI in patients with ACS.^29^ Pooled data from trials with this contemporary approach (newer generation drug-eluting stents and potent P2Y_12_i), confirm our findings: 1-month DAPT ranked first for reducing bleeding, whereas 3-month DAPT ranked first as the optimal period for reducing major ischemic events.^5^

At a molecular level, this temporal dissociation reflects the dynamic interplay between thrombogenic stimuli and platelet activation pathways after PCI in the ACS setting. In the early periprocedural phase, plaque disruption and subendothelial collagen exposure create a highly thrombogenic milieu that simultaneously activates multiple pathways—including thromboxane A_2_-dependent, ADP-dependent, and thrombin-mediated signaling^30^—making synergistic COX-1 and P2Y_12_ inhibition essential. As stent strut endothelialization progresses over the ensuing weeks^31^ and the acute prothrombotic stimulus subsides, the residual thrombotic risk transitions to an atherothrombotic substrate that can be effectively suppressed by potent P2Y12 inhibitors alone. In this later phase, the incremental antithrombotic benefit of aspirin becomes marginal, while its’ bleeding liability persists, thereby shifting the risk–benefit balance toward net clinical harm from continued dual therapy.^32^, ^33^, ^34^

The ongoing LEGACY trial (NCT05125276), designed to evaluate the impact of immediately omitting aspirin, and following with P2Y_12_i monotherapy, when compared with DAPT for 12 months on bleeding and ischemic events within 12 months following PCI in non–ST-elevation patients with ACS, will provide additional insights into this ultrashort DAPT approach, in addition to the observations of the NEO-MINDSET and STOPDAPT-3 trials, with a more selected population of non–ST-segment elevation acute coronary syndrome (NSTE-ACS). Similarly, the PREMIUM (NCT05709626) trial and other aspirin-free studies will further define the boundaries of safe practice in this clinical scenario,^35^ in which efficacy—namely reduction of early thrombotic events—seems to play a pivotal role in the first 30 days, while safety outcomes seems to outweigh such concerns beyond the first month in the patients enrolled in these 5 trials.

The heterogeneity in P2Y_12_i selection across the included trials (ticagrelor, prasugrel, and even clopidogrel in 1 trial) deserves consideration. While our analysis demonstrates consistent benefits across different agents, the potency and pharmacokinetic profiles of these medications vary considerably. Evidence from contemporary trials favors potent P2Y_12_i (ticagrelor and prasugrel) over clopidogrel for patients with ACS, particularly given concerns regarding clopidogrel’s reduced efficacy in certain patient populations^36^, ^37^, ^38^ and the greater scope of evidence with newer drugs, markedly ticagrelor.^5^

Nonetheless, our sensitivity analyses excluding the STOPDAPT-2 ACS trial, which used only clopidogrel as P2Y_12_i monotherapy, demonstrated consistent bleeding risk reduction while preserving ischemic protection. Notably, in the STOPDAPT-2 study, clopidogrel monotherapy failed to demonstrate noninferiority for the primary end point of NACE. Indeed, in a recent meta-analysis that demonstrated the safety and efficacy of short-term DAPT strategies, data from trials using ticagrelor were predominant. Thus, the choice of drug for single antiplatelet therapy after discontinuing aspirin should be cautiously evaluated and individualized, favoring potent drugs.

The absence of significant effect modification by ACS presentation type or diabetes status suggests that the bleeding benefit of early aspirin discontinuation is consistent across these clinical subgroups. Importantly, our sensitivity analyses using harmonized BARC bleeding definitions confirmed the robustness of the primary safety findings, mitigating concerns related to heterogeneity in end point adjudication across trials.

A key limitation of the reconstructed IPD approach is the inability to perform patient-level subgrouping for clinically actionable phenotypes such as ARC-HBR criteria, complex PCI features, or intravascular imaging-guided optimization, as these granular characteristics are not recoverable from published KM curves. Notably, the Sydney-2 IPD meta-analysis demonstrated that the bleeding benefit of P2Y12 inhibitor monotherapy was preserved irrespective of PCI complexity, without ischemic tradeoff^39^ and the TWILIGHT-HBR analysis showed that the absolute bleeding reduction with ticagrelor monotherapy was even more pronounced in patients meeting ARC-HBR criteria.^40^ These converging data suggest that our findings are likely generalizable to higher-risk procedural and clinical phenotypes.

Beyond the acute phase, optimal antiplatelet selection for chronic secondary prevention, beyond 12 months, deserves consideration. A recent IPD meta-analysis of 7 trials including nearly 29,000 patients with established coronary artery disease showed that clopidogrel monotherapy was superior to aspirin for long-term major adverse cardiac and cerebrovascular event prevention, without increasing major bleeding.^41^^,^^42^

From a clinical perspective, our findings support a nuanced approach to antiplatelet therapy selection that considers both patient-specific bleeding risk and the temporal evolution of thrombotic vulnerability following acute coronary interventions. The approximately 1-month DAPT duration evaluated across the included trials provides clinicians with evidence-based guidance for transitioning selected patients to simplified antiplatelet regimens while maintaining acceptable safety margins. This strategy, however, should be only considered in a scenario of contemporary interventional and antiplatelet strategies.

The implications for clinical practice guidelines merit careful consideration. Current European Society of Cardiology and American College of Cardiology/American Heart Association recommendations emphasize 12-month dual antiplatelet therapy duration for acute coronary syndrome patients undergoing percutaneous intervention, although shorter schemes are suggested for high bleeding risk individuals.^1^^,^^2^ Our findings, along with accumulating evidence from recent randomized trials, suggest that these recommendations may benefit from refinement to incorporate shorter duration strategies for appropriately selected patients with acceptable bleeding risk profiles and without complex anatomies and interventions that may increase the thrombotic risk (Central Illustration).

**Central Illustration:**
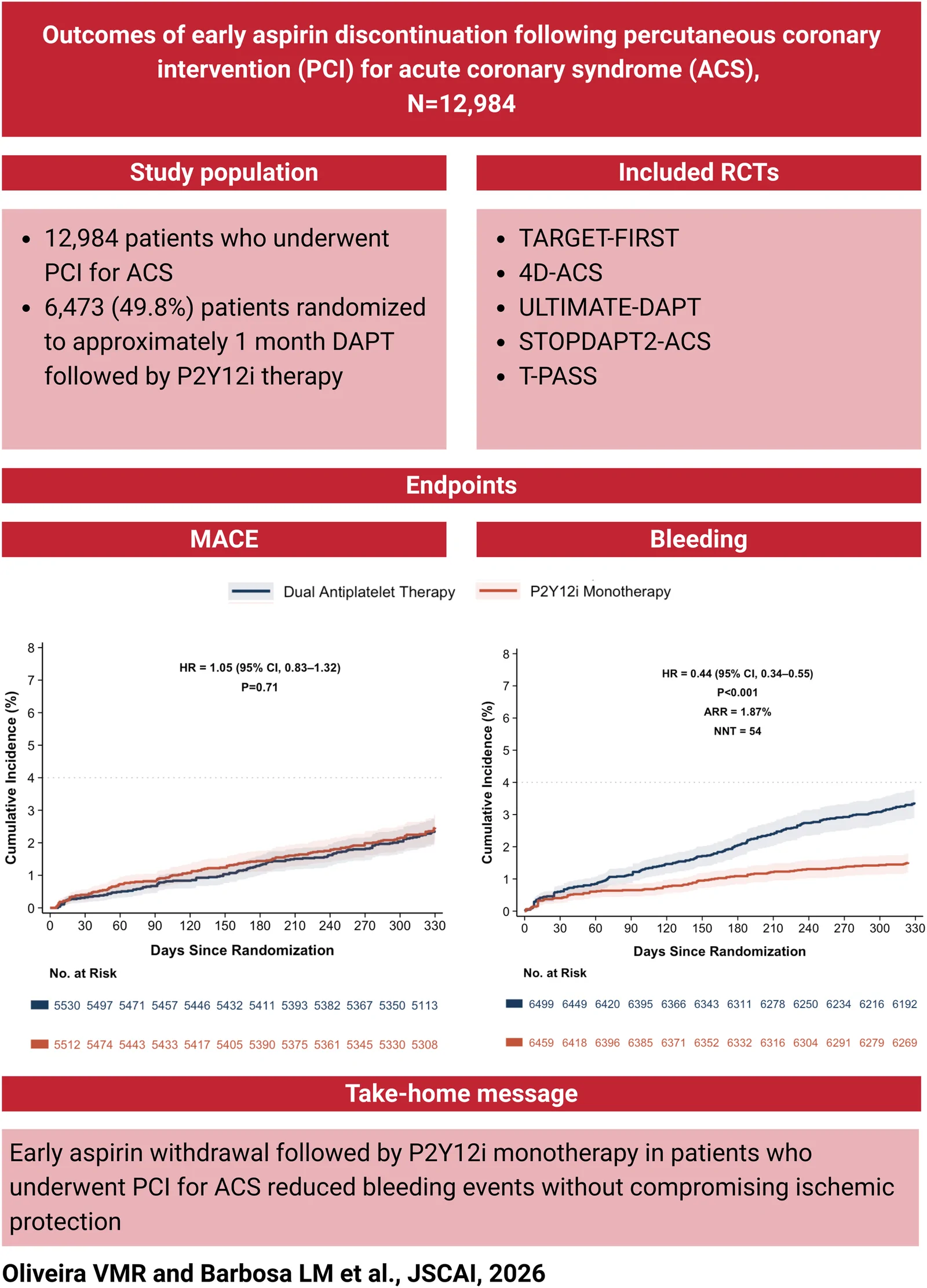
**Earlyaspirin discontinuation followed by P2Y**_**12**_**i monotherapy after percutaneous coronary intervention (PCI) for acute coronary syndrome (ACS) significantly reduced bleeding events without compromising ischemic protection across 5 randomized controlled trials.** ARR, absolute risk reduction; DCS, drug-coated stents; HR, hazard ratio; MACE, major adverse cardiovascular events; NNT, number needed to treat; STOPDAPT-2 ACS, Short and Optimal pciDuration of Dual Antiplatelet Therapy-2 Study for the Patients With Acute Coronary Syndrome; TARGET-FIRST, Evaluation of a Modified Antiplatelet Therapy Associated With Low-dose DES Firehawk in Acute Myocardial Infarction Patients Treated With Complete Revascularization Strategy; T-PASS, Ticagrelor Monotherapy in Patients Treated With New-Generation Drug-Eluting Stents for Acute Coronary Syndrome; ULTIMATE-DAPT, Comparison of 1 Month vs 12-month Dual Antiplatelet Therapy After Implantation of Drug-Eluting Stents Guided by Either Intravascular Ultrasound or Angiography in Patients With Acute Coronary Syndrome; 4D-ACS, The effect of short Duration of DAPT followed by Dose reduction after Implantation of DCS in ACS patients.

### Limitations

Several limitations warrant consideration in interpreting these results. The reconstruction of IPD from published KM curves, while methodologically rigorous, introduces potential for analytical artifacts that may not fully capture the complexity of the original datasets. Additionally, the heterogeneity in trial populations, P2Y_12_i selection, and baseline ischemic and bleeding risk profiles across the included studies may influence the generalizability of our findings. Furthermore, the eligible trials largely cluster around aspirin discontinuation at approximately 1 month (range, 16-39 days), which precludes the identification of a timing gradient or the determination of a precise optimal discontinuation time point. Accordingly, our findings should be interpreted as supporting the safety and efficacy of aspirin discontinuation at approximately 1 month, rather than as defining the ideal timing for this transition.

## Conclusions

In conclusion, this meta-analysis demonstrates that early aspirin discontinuation at approximately 1 month following PCI for ACS, with continuation of P2Y_12_i monotherapy, is a safe and effective strategy that significantly reduces bleeding without compromising ischemic protection in selected patients.

## CRediT authorship contribution statement

**Vinícius Martins Rodrigues Oliveira:** Conceptualization, Data curation, Methodology, Project administration, Software, Supervision, Writing – original draft, Writing – review & editing. **Lucas M. Barbosa:** Conceptualization, Data curation, Methodology, Writing – review & editing. **Ludimilla Pereira Tartuce:** Data curation, Writing – review & editing. **Robert Willian Anastácio Alcântara:** Data curation, Formal analysis, Writing – original draft. **Paulo Roberto Ferreira Tartuce Filho:** Data curation, Formal analysis. **Maria do Carmo Pereira Nunes:** Writing – review & editing. **Humberto Graner Moreira:** Supervision, Writing – original draft, Writing – review & editing. **Bruno Ramos Nascimento:** Methodology, Supervision, Writing – review & editing. **Deepak L. Bhatt:** Methodology, Supervision, Writing – review & editing.

## Declaration of competing interest

Deepak L. Bhatt discloses the following relationships—advisory board: Angiowave, Antlia Bioscience, Bayer, Boehringer Ingelheim, CellProthera, Cereno Scientific, E-Star Biotech, High Enroll, Janssen, Level Ex, McKinsey, Medscape Cardiology, Merck, NirvaMed, Novo Nordisk, Repair Biotechnologies, Stasys, SandboxAQ (stock options), and Tourmaline Bio; board of directors: American Heart Association New York City, Angiowave (stock options), Bristol Myers Squibb (stock), DRS.LINQ (stock options), and High Enroll (stock); consultant: Alnylam, Altimmune, Broadview Ventures, Corcept Therapeutics, Corsera, GlaxoSmithKline, Hims, Sanofi, SERB, SFJ, Summa Therapeutics, and Worldwide Clinical Trials; data monitoring committees: Acesion Pharma, Assistance Publique-Hôpitaux de Paris, Baim Institute for Clinical Research, Boston Scientific (Chair, PEITHO trial), Cleveland Clinic, Contego Medical (Chair, PERFORMANCE 2), Duke Clinical Research Institute, Mayo Clinic, Mount Sinai School of Medicine (for the ABILITY-DM trial, funded by Concept Medical; for ALLAY-HF, funded by Alleviant Medical), Novartis, Population Health Research Institute; and Rutgers University (for the NIH-funded MINT Trial); honoraria: American College of Cardiology (Senior Associate Editor, Clinical Trials and News, ACC.org; Chair, ACC Accreditation Oversight Committee), Arnold and Porter law firm (work related to Sanofi/Bristol Myers Squibb clopidogrel litigation), Baim Institute for Clinical Research (AEGIS-II executive committee funded by CSL Behring), Belvoir Publications (Editor in Chief, Harvard Heart Letter), Canadian Medical and Surgical Knowledge Translation Research Group (clinical trial steering committees), CSL Behring (AHA lecture), Duke Clinical Research Institute, Engage Health Media, HMP Global (Editor in Chief, Journal of Invasive Cardiology), Medtelligence/ReachMD (CME steering committees), MJH Life Sciences, Oakstone CME (Course Director, Comprehensive Review of Interventional Cardiology), Philips (Becker Webinar on AI), Population Health Research Institute, WebMD (CME steering committees), and Wiley (steering committee); other: Clinical Cardiology (Deputy Editor); Progress in Cardiovascular Diseases (Deputy Editor); patent: Sotagliflozin (named on a patent for sotagliflozin assigned to Brigham and Women’s Hospital who assigned to Lexicon; neither him nor Brigham and Women’s Hospital receive any income from this patent); research funding: Abbott, Acesion Pharma, Afimmune, Alnylam, Amarin, Amgen, AstraZeneca, Atricure, Bayer, Boehringer Ingelheim, Boston Scientific, CellProthera, Cereno Scientific, Chiesi, Cleerly, CSL Behring, Faraday Pharmaceuticals, Fractyl, Idorsia, Janssen, Javelin, Lexicon, Lilly, Medtronic, Merck, MiRUS, Moderna, Novartis, Novo Nordisk, Pfizer, PhaseBio, Regeneron, Reid Hoffman Foundation, Roche, Sanofi, Stasys, and 89Bio; royalties: Elsevier (Editor, Braunwald Heart Disease); and site co-investigator: Cleerly. Bruno Nascimento is supported in part by CNPq (Bolsa de produtividade em pesquisa, 310749/2022-0), by the Edwards Lifesciences Foundation (Improving the Prevention and Detection of Heart Valve Disease Across the Lifespan, 2026) and by FAPEMIG (grant APQ-03291-18). Vinícius Oliveira reports receiving a travel grant from Fundação de Amparo à Pesquisa do Estado de Goiás (FAPEG) to present this work at EuroPCR 2026. Lucas Barbosa, Ludimilla Tartuce, Robert Alcântara, Paulo Filho, Maria Nunes, Humberto Moreira, and Bruno Nascimento report no relationships that could be construed as a conflict of interest. All authors take responsibility for all aspects of the reliability and freedom from bias of the presented data and their discussed interpretation.

## Footnotes

To access the supplementary material accompanying this article, visit the online version of the *Journal of the Society for Cardiovascular Angiography & Interventions* at 10.1016/j.jscai.2026.105456.
