# Supplementary material for: Early Aspirin Discontinuation Following PCI for Acute Coronary Syndromes: A Systematic Review and Meta-Analysis of Reconstructed Individual Patient Data
Source: J Soc Cardiovasc Angiogr Interv. 2026 May 20;5(9):105456. doi: 10.1016/j.jscai.2026.105456 (PMC13591527; doi:10.1016/j.jscai.2026.105456)
Supplement: Supplementary file 1 [file mmc1.docx]

**Early Aspirin Discontinuation Following PCI for Acute Coronary Syndromes: A Systematic Review and Meta-Analysis of Reconstructed Individual Patient Data**

Vinícius Martins Rodrigues Oliveira^1^, MS *, Lucas M. Barbosa^2^, MS *, Ludimilla Pereira Tartuce, MD, MSc^3^, Robert Willian Anastácio Alcântara, MD^4^, Paulo Roberto Tartuce Filho, MD³, Maria do Carmo Pereira Nunes, MD, PhD^2^, Humberto Graner Moreira, MD, PhD, FESC^1,5^ Bruno Ramos Nascimento, MD, PhD, FACC, FESC^2 †^, Deepak L. Bhatt, MD, MPH, MBA^6 †^

*¹Faculdade de Medicina, Universidade Federal de Goiás, Goiânia, Brazil
²Federal University of Minas Gerais, Belo Horizonte, Brazil
³Instituto de Cardiologia e Radiologia Intervencionista de Rio Verde, Rio Verde, Brazil
^4^Clínica Aurus, Oliveira, Brazil
^5^Einstein Hospital Israelita, São Paulo, Brazil
^6^Mount Sinai Fuster Heart Hospital, New York, The United States of America*

**Both authors contributed equally as co-first authors*

*†Both authors contributed equally as co-senior authors*

**Table of Contents**

Supplementary Methods 1. PRISMA Checklist	4

Supplementary Methods 2. PRISMA Abstract Checklist……………………………………………..…………..10

Supplemental Methods 3. Details of the Search Strategy	11

Supplemental Table S1 . Main Inclusion and Exclusion Criteria Used by Study………….………..12

Supplemental Table S2.. Composite endpoints definitions for each study and timing of aspirin removal and randomization……………………………………………………………….…………14

***Supplemental Figure S1. Landmark analyses for the endpoint of MACE………………………..17
Supplemental Figure S2. Landmark analyses for the endpoint of bleeding………………………19***

***Supplemental Figure S3. Forest plots for secondary the harmonized definition of major bleeding (BARC 3 or 5)……………….21***

***Supplemental Figure S4. Forest plots for secondary the harmonized definition of clinically relevant bleeding (BARC 2,3 or 5)……………….21***

***Supplemental Figure S5. Sensitivity analyses for the endpoints of MACE and bleeding excluding the STOPDAPT-2 ACS study..22***

***Supplemental Figure S6. Forest plots for secondary endpoints…………………………….24***

***Supplemental Figure S7. Exploratory subgroup analyses for the endpoint of MACE according to ………………………..27***

***Supplemental Figure S8. Exploratory subgroup analyses for the endpoint of bleeding according to the status of………..30***

***Supplement Figure S9. Sensitivity analyses using Mantel-Haenszel with published hazard ratios for the endpoints analyzed through KM reconstruction…………………33***

***Supplemental Figure S10. Risk of bias in the included studies………………………………………………………………………………………………………………34***

**Supplement Table S3. Comparison of published versus reconstructed hazard ratios for each endpoint………………………………………………………………………………35**

**Supplemental Table S4. Grading of Recommendations, Assessment, Development and Evaluations (GRADE)………………………………………………………………………..36**

**Supplemental Table S5. Crude outcomes per study for the endpoints of MACE, bleeding and NACE……………….37**

**Supplemental Methods 1.** PRISMA 2020 Main Checklist

| **Topic** | **No.** | | **Item** | **Location where item is reported** |
| --- | --- | --- | --- | --- |
| **TITLE** | |  |  |  |
| **Title** | | 1 | Identify the report as a systematic review. | Pg. 1 at MS |
| **ABSTRACT** | |  |  |  |
| **Abstract** | | 2 | See the PRISMA 2020 for Abstracts checklist | NA |
| **INTRODUCTION** | |  |  |  |
| **Rationale** | | 3 | Describe the rationale for the review in the context of existing knowledge. | Pg. 3 at MS |
| **Objectives** | | 4 | Provide an explicit statement of the objective(s) or question(s) the review addresses. | Pg. 3 at MS |
| **METHODS** | |  |  |  |
| **Eligibility criteria** | | 5 | Specify the inclusion and exclusion criteria for the review and how studies were grouped for the syntheses. | Pg. 7-8 at MS |
| **Information sources** | | 6 | Specify all databases, registers, websites, organisations, reference lists and other sources searched or consulted to identify studies. Specify the date when each source was last searched or consulted. | Pg. 7 at MS |
| **Search strategy** | | 7 | Present the full search strategies for all databases, registers and websites, including any filters and limits used. | Pg. 11 at sup |
| **Selection process** | | 8 | Specify the methods used to decide whether a study met the inclusion criteria of the review, including how many reviewers screened each record and each report retrieved, whether they worked independently, and if applicable, details of automation tools used in the process. | Pg. 7-8 at MS |
| **Data collection process** | | 9 | Specify the methods used to collect data from reports, including how many reviewers collected data from each report, whether they worked independently, any processes for obtaining or confirming data from study investigators, and if applicable, details of automation tools used in the process. | Pg. 7-9 at MS |
| **Data items** | | 10a | List and define all outcomes for which data were sought. Specify whether all results that were compatible with each outcome domain in each study were sought (e.g. for all measures, time points, analyses), and if not, the methods used to decide which results to collect. | Pg. 8-9 at MS |
|  | | 10b | List and define all other variables for which data were sought (e.g. participant and intervention characteristics, funding sources). Describe any assumptions made about any missing or unclear information. | Pg. 8-9 at MS |
| **Study risk of bias assessment** | | 11 | Specify the methods used to assess risk of bias in the included studies, including details of the tool(s) used, how many reviewers assessed each study and whether they worked independently, and if applicable, details of automation tools used in the process. | Pg. 8-9 at MS |
| **Effect measures** | | 12 | Specify for each outcome the effect measure(s) (e.g. risk ratio, mean difference) used in the synthesis or presentation of results. | Pg. 9 at MS |
| **Synthesis methods** | | 13a | Describe the processes used to decide which studies were eligible for each synthesis (e.g. tabulating the study intervention characteristics and comparing against the planned groups for each synthesis (item 5). | Table 1 |
|  | | 13b | Describe any methods required to prepare the data for presentation or synthesis, such as handling of missing summary statistics, or data conversions. | Pg. 7-9 at MS |
|  |  | 13c | Describe any methods used to tabulate or visually display results of individual studies and syntheses. | Pg. 9-10 at MS |
|  |  | 13d | Describe any methods used to synthesize results and provide a rationale for the choice(s). If meta-analysis was performed, describe the model(s), method(s) to identify the presence and extent of statistical heterogeneity, and software package(s) used. | Pg. 9-10 at MS |
|  |  | 13e | Describe any methods used to explore possible causes of heterogeneity among study results (e.g. subgroup analysis, meta-regression). | Pg. 9-10 at MS |
|  |  | 13f | Describe any sensitivity analyses conducted to assess robustness of the synthesized results. | Pg. 9-10 at MS |
| **Reporting bias assessment** | | 14 | Describe any methods used to assess risk of bias due to missing results in a synthesis (arising from reporting biases). | Pg. 9 at MS |
| **Certainty assessment** | | 15 | Describe any methods used to assess certainty (or confidence) in the body of evidence for an outcome. | NA |
| **RESULTS** | |  |  |  |
| **Study selection** | | 16a | Describe the results of the search and selection process, from the number of records identified in the search to the number of studies included in the review, ideally using a flow diagram. | Figure 1 |
|  | | 16b | Cite studies that might appear to meet the inclusion criteria but which were excluded, and explain why they were excluded. | NA |
| **Study characteristics** | | 17 | Cite each included study and present its characteristics. | Table 1, Sup. Tables 1-2 |
| **Risk of bias in studies** | | 18 | Present assessments of risk of bias for each included study. | Pg. 14 at MS, Sup. Fig. 10 (Pg. 34 at Sup.) |
| **Results of individual studies** | | 19 | For all outcomes, present, for each study: (a) summary statistics for each group (where appropriate) and (b) an effect estimate and its precision (e.g., confidence/credible interval), ideally using structured tables or plots. | Fig. 2-3 at MS, Sup. Figs. 7-10 |
| **Results of syntheses** | | 20a | For each synthesis, briefly summarise the characteristics and risk of bias among contributing studies. | Supplemental Figure 10 |
|  | | 20b | Present the results of all statistical syntheses conducted. If meta-analysis was done, present for each the summary estimate and its precision (e.g., confidence/credible interval), and measures of statistical heterogeneity. If comparing groups, describe the direction of the effect. | Fig. 2-3 at MS, Sup. Figs. 1-9 |
|  |  | 20c | Present results of all investigations of possible causes of heterogeneity among study results. | Pg. 12-14 at MS, Sup. Figs. 8-9, Sup. Table 3. |
|  |  | 20d | Present all sensitivity analyses conducted to assess the robustness of the synthesized results. | Sup. Figs. 1,-2, 7-9 |
| **Reporting biases** | | 21 | Present assessments of risk of bias due to missing results (arising from reporting biases) for each synthesis assessed. | Sup. Fig. 10 |
| **Certainty of evidence** | | 22 | Present assessments of certainty (or confidence) in the body of evidence for each outcome assessed. | NA |
| **DISCUSSION** | |  |  |  |
| **Discussion** | | 23a | Provide a general interpretation of the results in the context of other evidence. | Pg. 15-21 at MS |
|  | | 23b | Discuss any limitations of the evidence included in the review. | Pg. 21-22 at MS |
|  |  | 23c | Discuss any limitations of the review processes used. | Pg. 21-22 at MS |
|  |  | 23d | Discuss the implications of the results for practice, policy, and future research. | Pg. 21-22 at MS |
| **OTHER INFORMATION** | |  |  |  |
| **Registration and protocol** | | 24a | Provide registration information for the review, including the register name and registration number, or state that the review was not registered. | PROSPERO 2025 CRD420251144691 |
|  | | 24b | Indicate where the review protocol can be accessed or state that a protocol was not prepared. | https://www.crd.york.ac.uk/PROSPERO/view/CRD420251144691 |
|  |  | 24c | Describe and explain any amendments to information provided at registration or in the protocol. | None. |
| **Support** | | 25 | Describe sources of financial or non-financial support for the review and the role of the funders or sponsors. | None |
| **Competing interests** | | 26 | Declare any competing interests of review authors. | Pg. 2-3 at MS |
| **Availability of data, code, and other materials** | | 27 | Report which of the following are publicly available and where they can be found template data collection forms, data extracted from included studies, data used for all analyses, analytic code, and any other materials used in the review. | Non-Available |

^Abbreviations: MS, manuscript; sup., supplement.^

**Supplementary Methods 2. PRISMA Abstract Checklist**

| **Topic** | **No.** | **Item** | **Reported?** |
| --- | --- | --- | --- |
| **TITLE** |  |  |  |
| **Title** | 1 | Identify the report as a systematic review. | Yes |
| **BACKGROUND** |  |  |  |
| **Objectives** | 2 | Provide an explicit statement of the main objective(s) or question(s) the review addresses. | Yes |
| **METHODS** |  |  |  |
| **Eligibility criteria** | 3 | Specify the inclusion and exclusion criteria for the review. | Yes |
| **Information sources** | 4 | Specify the information sources (e.g., databases, registers) used to identify studies and the date each was last searched. | Yes |
| **Risk of bias** | 5 | Specify the methods used to assess the risk of bias in the included studies. | No |
| **Synthesis of results** | 6 | Specify the methods used to present and synthesize results. | Yes |
| **RESULTS** |  |  |  |
| **Included studies** | 7 | Give the total number of included studies and participants and summarise relevant characteristics of studies. | Yes |
| **Synthesis of results** | 8 | Present results for main outcomes, preferably indicating the number of included studies and participants for each. If meta-analysis was done, report the summary estimate and confidence/credible interval. If comparing groups, indicate the direction of the effect (i.e., which group is favored). | Yes |
| **DISCUSSION** |  |  |  |
| **Limitations of evidence** | 9 | Provide a brief summary of the limitations of the evidence included in the review (e.g. study risk of bias, inconsistency and imprecision). | No |
| **Interpretation** | 10 | Provide a general interpretation of the results and important implications. | No |
| **OTHER** |  |  |  |
| **Funding** | 11 | Specify the primary source of funding for the review. | No |
| **Registration** | 12 | Provide the register name and registration number. | No |
|  |  |  |  |

# Supplemental Methods 3. Details of the Search Strategy

| Database | Search strategy |
| --- | --- |
| PubMed | ("aspirin" OR "dual antiplatelet therapy" OR "DAPT") AND ("acute coronary syndrome" OR "ACS" OR "STEMI" OR "myocardial infarction") AND ("percutaneous coronary intervention" OR "PCI") AND (randomized controlled trial[pt] OR controlled clinical trial[pt] OR clinical trials as topic[mesh:noexp] OR trial[ti] OR random*[tiab] OR placebo*[tiab]) |
| Embase | ('aspirin'/exp OR 'aspirin' OR 'dual antiplatelet therapy'/exp OR 'dual antiplatelet therapy' OR 'dapt'/exp OR 'dapt') AND ('acute coronary syndrome'/exp OR 'acute coronary syndrome' OR 'acs'/exp OR 'acs' OR 'stemi'/exp OR 'stemi' OR 'myocardial infarction'/exp OR 'myocardial infarction') AND ('percutaneous coronary intervention'/exp OR 'percutaneous coronary intervention' OR 'pci') AND ('randomized controlled trial'/exp OR 'randomized controlled trial') |
| Cochrane | ("aspirin" OR "dual antiplatelet therapy" OR "DAPT") AND ("acute coronary syndrome" OR "ACS" OR "STEMI" OR "myocardial infarction") AND ("percutaneous coronary intervention" OR "PCI") AND (randomized controlled trial[pt] OR controlled clinical trial[pt] OR clinical trials as topic[mesh:noexp] OR trial[ti] OR random*[tiab] OR placebo*[tiab]) |

# Supplemental Table S1. Main Inclusion and Exclusion Criteria Used by Study

| Study | Inclusion criteria | Exclusion criteria |
| --- | --- | --- |
| ULTIMATE-DAPT | Patients aged 18 years or older; had an acute coronary syndrome (ie, unstable angina [angiography showing a severely narrowed or ruptured plaque or thrombotic lesion without cardiac biomarker elevation], non-ST segment elevation myocardial infarction [NSTEMI], or ST-segment elevation myocardial infarction [STEMI]) caused by a culprit lesion in an untreated coronary artery segment, up to 30 days before randomisation;18 and had an indication for percutaneous coronary intervention with a second-generation drug- eluting stent | Patients with stroke within 3 months or any permanent neurological deficit; any previous intracranial bleed or intracranial disease (eg, aneurysm or fistula); previous coronary artery bypass graft surgery; any planned surgery within 12 months; any reason for which antiplatelet therapy might need to be discontinued within 12 months; severe chronic kidney disease (defined as an estimated glomerular filtration rate <20 mL/min per 1·73 m²); need for chronic oral anticoagulation (ie, warfarin or coumadin or direct oral anticoagulants); a platelet count of less than 100 000 mm³; contraindication to aspirin or ticagrelor;  liver cirrhosis; people intending to become pregnant; a life expectancy of less than 1 year; and any condition likely to interfere with study processes, including medication compliance or follow-up visits (eg, dementia, alcohol abuse, severe frailty, or required to travel a long distance for follow-up visits). |
| T-PASS | Patients that had an ACS and had undergone DES implantation with a biodegradable polymer sirolimus-eluting stent (Orsiro, Biotronik, Switzerland). ACS included myocardial infarction (with or without electrocardiographic evidence of ST-segment elevation) and unstable angina, defined as typical symptoms such as recurrent chest pain at rest or with minimal exertion and manifestation of severe angina initiated or aggravated within 4 weeks before the index procedure.. | Patients that had increased risk of bleeding such as anemia and thrombocytopenia, and a need for oral anticoagulation therapy. |
| 4D-ACS | Patients were eligible for inclusion if they had ACS, including myocardial infarction (MI) – defined according to the Fourth Universal Definition of MI – or unstable angina, characterised by recurrent chest pain at rest or with minimal exertion, or new or worsening severe angina within 4 weeks before the index procedure, and if they underwent PCI with a biolimus-coated stent (BioFreedom, Ultra [Biosensors]) | Patients who required anticoagulant therapy or had a history of cerebral infarction were excluded from the study. |
| TARGET-FIRST | Patients were eligible for enrollment if they were at least 18 years of age, were hospitalized for ST-segment elevation myocardial infarction (STEMI) or non-STEMI (NSTEMI), and had undergone complete revascularization with an abluminal biodegradable-polymer rapamycin-eluting stent (Firehawk Liberty, MicroPort) without complications. Complete revascularization was defined as treatment of all clinically significant lesions (as determined on angiography) during the index or staged (within 7 days after myocardial infarction) PCI procedure. The final decision regarding the lesion treatment strategy was at the investigator’s discretion. | Patients were excluded if they had a high risk of bleeding (i.e., if they had secondary fibrinolysis, were receiving oral anticoagulant therapy, had an estimated glomerular filtration rate of <30 ml per minute per 1.73 m2 of body-surface area or were undergoing dialysis, had active bleeding, had a pre-PCI hemoglobin level of <13 g per deciliter [men] or <12 g per deciliter [women], had liver cirrhosis, had a history of bleeding diathesis, or had thrombocytopenia). Patients were also excluded if they had a high risk of an ischemic event (i.e., if they had undergone complex PCI, had a chronic total occlusion, had left main coronary artery disease, had received stents that totaled >80 mm in length, or had severe calcification). |
| STOPDAPT-2 ACS | Patients who underwent successful PCI for ACS with CoCr-EES (Xience Series, Abbott Vascular) without concomitant use of other types of drug-eluting stent or in-hospital major complications other than periprocedural MI. | Key exclusion criteria are limited to continued use of oral anticoagulants and previous history of hemorrhagic stroke. |

# Supplemental Table S2. Composite endpoints definitions for each study and timing of aspirin removal and randomization

| **Study** | **MACCE** | **NACE** | **Major bleeding** | **Clinically relevant bleeding** | **Aspirin removal** | **Timing of randomization** |
| --- | --- | --- | --- | --- | --- | --- |
| STOPDAPT-2 ACS | Cardiovascular death, myocardial infarction [MI], any stroke, or definite stent thrombosis | - | - | TIMI major or minor bleeding | 1–2 months (median 39 days) | Median of 5 days after PCI |
| T-PASS | Composite of all-cause death, myocardial infarction, definite or probable stent thrombosis and stroke | Composite of all-cause death, myocardial infarction, definite or probable stent thrombosis, stroke, and major bleeding at 1 year after the index procedure | BARC 3 to 5 | - | <1 month (median 16 days) | At PCI |
| TARGET FIRST | composite of death from any cause, myocardial infarction, stent thrombosis, stroke, | composite of death from any cause, myocardial infarction, stent thrombosis, stroke, or major bleeding (defined by the Bleeding Academic Research Consortium [BARC] as a bleeding event of type 3 or 5) | - | BARC 2,3 or 5 | 1 month post-PCI | 1 month after PCI |
| 4D-ACS | composite of death, non-fatal myocardial infarction, stroke, ischaemia-driven target vessel revascularisation, | composite of death, non-fatal myocardial infarction, stroke, ischaemia-driven target vessel revascularisation, and Bleeding Academic Research Consortium Type 2-5 bleeding. | BARC 3 to 5 | - | 1 month post-PCI | At PCI |
| ULTIMATE-DAPT | composite of cardiac death, myocardial infarction, ischaemic stroke, definite stent thrombosis, or  clinically driven target vessel revascularisation | - | - | BARC 2,3 or 5 | 1 month post-PCI | 1st randomization at PCI  2^nd^ randomization 1 month post-PCI (at aspirin discontinuation) |

BARC: Bleeding Academic Research Consortium; Myocardial Infarction

**Supplemental Figure S1. Landmark analyses for the endpoint of MACE**

**A)**

**B)**

**Supplemental Figure S2. Landmark analyses for the endpoint of bleeding**

**A)**

**B)**

**Supplemental Figure S3. Forest plots for secondary the harmonized definition of major bleeding (BARC 3 or 5)**

**Supplemental Figure S4. Forest plots for secondary the harmonized definition of clinically relevant bleeding (BARC 2,3 or 5)**

**Supplemental Figure S5. Sensitivity analyses for the endpoints of MACE and bleeding excluding the STOPDAPT-2 ACS study.**

A- **Bleeding**

**B – MACE**

**Supplemental Figure S6. Forest plots for secondary endpoints**

A) **All-cause death**

B) **Myocardial infarction**

C) **Stent thrombosis**

D) **Stroke**

E) **Target vessel revascularization**

**Supplemental Figure S7. Exploratory subgroup analyses for the endpoint of MACE according to**

A) **ACS presentation type**

B) **Diabetes Mellitus**

C) **Multivessel disease**

**Supplemental Figure S8. Exploratory subgroup analyses for the endpoint of bleeding according to the status of**

A) **ACS presentation type**

B) **Diabetes Mellitus**

C) **Multivessel disease**

**Supplement Figure S9. Sensitivity analyses using Mantel-Haenszel with published hazard ratios for the endpoints analyzed through KM reconstruction**

A) **MACE**

B) **Bleeding**

C) **NACE**

**Supplemental Figure S10. Risk of bias in the included studies**

**Supplement Table S3. *Comparison of published versus reconstructed hazard ratios for each endpoint***

| **Endpoint** | **Published HR (95% CI)** | **Reconstructed HR (95% CI)** |
| --- | --- | --- |
| MACE | 1.03 (0.76–1.40) | 1.05 (0.83–1.32) |
| Bleeding | 0.44 (0.28–0.70) | 0.44 (0.34–0.55) |
| NACE* | 0.70 (0.50–0.99) | 0.74 (0.63–0.88) |

** TARGET-FIRST did not publish an HR for NACE; Kaplan-Meier curves were reconstructed for inclusion in the IPD pooled analysis.*

**Supplemental Table S4. Grading of Recommendations, Assessment, Development and Evaluations**

| **P2Y12i monotherapy compared to DAPT for post PCI for ACS**  **Bibliography:** | | | | | | | | | | | |
| --- | --- | --- | --- | --- | --- | --- | --- | --- | --- | --- | --- |
| **Certainty assessment** | | | | | | | **Summary of findings** | | | | |
| **Participants (studies) Follow-up** | **Risk of bias** | **Inconsistency** | **Indirectness** | **Imprecision** | **Publication bias** | **Overall certainty of evidence** | **Study event rates (%)** | | **Relative effect (95% CI)** | **Anticipated absolute effects** | |
|  |  |  |  |  |  |  | **With DAPT** | **With P2Y12i monotherapy** |  | **Risk with DAPT** | **Risk difference with P2Y12i monotherapy** |
| **Major Adverse Cardiac Events (assessed with: HR)** | | | | | | | | | | | |
| 11042 (4 RCTs) | not serious | not serious | serious^a^ | not serious | none | ⨁⨁⨁◯ Moderate^a^ | 5530 participants | 5512 participants | **HR 1.05** (0.83 to 1.32) [Major Adverse Cardiac Events] | **Low** | |
|  |  |  |  |  |  |  |  |  |  | 29 per 1,000 | **1 more per 1,000** (from 5 fewer to 9 more) |
| **Bleeding (assessed with: HR)** | | | | | | | | | | | |
| 12958 (5 RCTs) | not serious | not serious | serious^b^ | not serious | none | ⨁⨁⨁◯ Moderate^b^ | 6499 participants | 6459 participants | **HR 0.44** (0.34 to 0.55) [Bleeding] | **Low** | |
|  |  |  |  |  |  |  |  |  |  | 34 per 1,000 | **19 fewer per 1,000** (from 22 fewer to 15 fewer) |
| **NACE** | | | | | | | | | | | |
| 12984 (5 RCTs) | not serious | not serious | serious^b^ | not serious | none | ⨁⨁⨁◯ Moderate^b^ | 6511 participants | 6473 participants | **HR 0.74** (0.63 to 0.88) [NACE] | **Low** | |
|  |  |  |  |  |  |  |  |  |  | 49 per 1,000 | **13 fewer per 1,000** (from 18 fewer to 6 fewer) |
| **Major bleeding** | | | | | | | | | | | |
| 12984 (2 RCTs) | not serious | not serious | not serious | not serious | none | ⨁⨁⨁⨁ High | 6511 participants | 6473 participants | **HR 0.40** (0.26 to 0.61) [Major bleeding] | **Low** | |
|  |  |  |  |  |  |  |  |  |  | 50 per 1,000 | **30 fewer per 1,000** (from 37 fewer to 19 fewer) |
| **Clinically relevant bleeding** | | | | | | | | | | | |
| 8848 (3 RCTs) | not serious | not serious | not serious | not serious^b^ | none | ⨁⨁⨁⨁ High^b^ | 4433 participants | 4415 participants | **HR 0.43** (0.34 to 0.55) [Clinically relevant bleeding] | **Low** | |
|  |  |  |  |  |  |  |  |  |  | 41 per 1,000 | **23 fewer per 1,000** (from 27 fewer to 18 fewer) |

**CI:** confidence interval; **HR:** hazard ratio

#### Explanations

a. The endpoint definition varies across trials

b. The endpoint definition varies across trials (BARC and TIMI)

**Supplemental Table S5. Crude outcomes per study for the endpoints of MACE, bleeding and NACE**

**MACE**

| Study | P2Y12i monotherapy | DAPT |
| --- | --- | --- |
| TARGET-FIRST | 15 (1.6) | 16 (1.6) |
| 4D-ACS | 8 (2.4) | 12 (3.7) |
| STOPDAPT-2 ACS | 56 (2.7) | 38 (1.9) |
| ULTIMATE DAPT | 61 (3.6) | 63 (3.7) |
| T-PASS | 21 (1.5) | 31 (2.2) |

BLEEDING

| Study | P2Y12i monotherapy | DAPT |
| --- | --- | --- |
| TARGET-FIRST | 25 (2.6) | 54 (2.6) |
| 4D-ACS | 2 (0.6) | 15 (4.6) |
| STOPDAPT-2 ACS | 11(0.5) | 24 (1.2) |
| ULTIMATE DAPT | 35 (2.1) | 78 (4.6) |
| T-PASS | 17 (1.2) | 48 (3.4) |

NACE

| Study | P2Y12i monotherapy | DAPT |
| --- | --- | --- |
| TARGET-FIRST | 20 (2.1) | 21 (2.2) |
| 4D-ACS | 16 (4.9) | 29 (8.8) |
| STOPDAPT-2 ACS | 65 (3.2) | 58 (2.8) |
| ULTIMATE DAPT | 97 (5.7) | 140 (8.2) |
| T-PASS | 40 (2.8) | 73 (5.2) |

Numbers are expressed as n (%); P2Y12i: P2Y12 inhibitors; DAPT: Dual antiplatelet therapy
